# A low-cost automated growth chamber system for continuous measurements of gas exchange at canopy scale in dynamic conditions

**DOI:** 10.1186/s13007-021-00772-z

**Published:** 2021-06-30

**Authors:** Nicole Salvatori, Alberti Giorgio, Onno Muller, Uwe Rascher, Alessandro Peressotti

**Affiliations:** 1 Department of Life Sciences, University of Trieste, 34127 Trieste, Italy; 2 Department of Agricultural, Food, Environmental and Animal Sciences, University of Udine, Via delle Scienze 206, 33100 Udine, Italy; 3 Institute of Bio- and Geosciences, IBG-2: Plant Sciences, Forschungszentrum Jülich GmbH, Leo-Brandt-Str, 52425 Jülich, Germany

**Keywords:** Growth chamber, Canopy, Low-cost, Fluctuating light, Dynamic photosynthesis

## Abstract

**Background:**

Obtaining instantaneous gas exchanges data is fundamental to gain information on photosynthesis. Leaf level data are reliable, but their scaling up to canopy scale is difficult as they are acquired in standard and/or controlled conditions, while natural environments are extremely dynamic. Responses to dynamic environmental conditions need to be considered, as measurements at steady state and their related models may overestimate total carbon (C) plant uptake.

**Results:**

In this paper, we describe an automatic, low-cost measuring system composed of 12 open chambers (60 × 60 × 150 cm; around 400 euros per chamber) able to measure instantaneous CO_2_ and H_2_O gas exchanges, as well as environmental parameters, at canopy level. We tested the system’s performance by simulating different CO_2_ uptake and respiration levels using a tube filled with soda lime or pure CO_2_, respectively, and quantified its response time and measurement accuracy. We have been also able to evaluate the delayed response due to the dimension of the chambers, proposing a method to correct the data by taking into account the response time (${t}_{0}$) and the residence time (τ). Finally, we tested the system by growing a commercial soybean variety in fluctuating and non-fluctuating light, showing the system to be fast enough to capture fast dynamic conditions. At the end of the experiment, we compared cumulative fluxes with total plant dry biomass.

**Conclusions:**

The system slightly over-estimated (+ 7.6%) the total C uptake, even though not significantly, confirming its ability in measuring the overall CO_2_ fluxes at canopy scale. Furthermore, the system resulted to be accurate and stable, allowing to estimate the response time and to determine steady state fluxes from unsteady state measured values. Thanks to the flexibility in the software and to the dimensions of the chambers, even if only tested in dynamic light conditions, the system is thought to be used for several applications and with different plant canopies by mimicking different environmental conditions.

**Supplementary Information:**

The online version contains supplementary material available at 10.1186/s13007-021-00772-z.

## Background

Despite being the most important biological process on Earth, photosynthesis still presents mechanisms that are not deeply understood and it is considered a matter of priority interest for new pioneering research fields [5, 45]. By converting solar energy into chemical energy, plants accumulate biomass by which several human activities depend on, as food, fodder, litter and fuelwood [12, 49]. Due to the rise in food demands [2, 44] and, more general, in plant-derived products, the newest research is aiming to target those processes in photosynthesis that would improve the overall crop yield [22, 23, 33]. This can be achieved in laboratories and tested in green houses where, however, it is difficult to mimic real field conditions. In fact, in natural environments, plants are affected simultaneously by several abiotic conditions (i.e. changes in temperature, light intensity, humidity) and biological interactions, which could translate into uncertainties in the experimental results [4, 20].

To facilitate the translation of information from the laboratory to the field, it is also necessary to mimic natural environmental conditions within growth chambers [15]. For example, simulating dynamic light conditions is necessary to retrieve canopy scale data that would reflect environmental variability [4]. In fact, whereas most of the past experiments and models considered photosynthesis at the steady state [10, 11, 17, 43, 48, 53], the importance of considering some photosynthetic processes in their transient states has been recognized [9, 14, 31, 46, 47]. Plants are exposed to fluctuating irradiance due to the movements of clouds, the effect of wind and the gaps within the canopy [35, 39]. How plants respond to these dynamic conditions affects carbon dioxide (CO_2_) uptake and final biomass yield. Plants can adjust to the dynamic environmental conditions by regulating the stomata [8, 26, 37], by moving their chloroplasts within the leaves or by moving their leaves within the canopy [16], by regulating photochemical properties [14], by activating Calvin Cycle enzymes and by controlling photo-protective processes [41]. Therefore, continuous measurements of gas exchanges are necessary to unravel the effects of dynamic environmental conditions on plants.

Gas exchange methods at leaf level are usually based on a leaf cuvette connected to an Infrared Gas Analyser (IRGA) measuring the difference among external and internal CO_2_ concentration (closed systems) or between the inlet and the outlet air (open systems). These methods allow the estimation of several physiological parameters such as, for example, net photosynthesis and stomatal conductance [21, 28]. When gas exchange measurements are combined with chlorophyll fluorescence, several other parameters related to photochemistry and the primary reactions of photosynthesis (i.e. light-harvesting and energy dissipation) can be retrieved [6, 27]. Leaf level data are reliable and repeatable, but these data can be hardly scaled up at whole plant or whole canopy scale, in particular in dynamic conditions, unless using cross-scale modelling [52].

Growth chamber systems allow direct CO_2_ gas exchange measurements at plant or small canopy scales. In open chambers, net carbon (C) exchange is estimated by measuring the inlet flux and the difference between inlet and outlet CO_2_ concentrations; in closed chambers, the change with time in CO_2_ concentration within the chamber headspace is measured and the assimilation rate is then calculated [12, 51]. While open chambers can measure gas exchange for long time periods, closed chambers can be used only for short time periods in order to avoid increase in air temperature or water condensation [24]. Several growth chamber systems have been described in the literature [3, 12, 30], but some of them showed low ability to control environmental conditions [29, 42], are not adapted to long-term continuous measurements [3] or are rather expensive (see [54] for a comprehensive review of space growth chambers).

Besides of the growth chamber systems, other systems have been developed in the last decades such as phytotrones [19] and the ‘exotic’ Biosphere 2 Laboratory [38], with the idea of allowing complete control of environmental variables [19] and the scaling up of the measured values from the laboratory to model ecosystems [32]. Nevertheless, even if relevant tests have been performed, the conditions found within these systems are often dissimilar to natural conditions that it is, again, difficult to relate these results to field data [20].

On the other hand, canopy gas exchange measurements can be continuously measured in the field using micro-meteorological techniques, such as eddy covariance [7, 25, 50]. These systems have been demonstrated to be reliable even though can be used only in specific site conditions (i.e. flat terrain, large footprint areas, atmospheric stability,[1]). Moreover, as several abiotic factors can simultaneously change in the field (i.e. light, temperature, humidity, etc.), it is then difficult to isolate the effects of the fluctuations of each single factor on instantaneous C exchanges at such a scale. Therefore, it is relevant to design growth chamber for continuous gas exchange measurements able to control different environmental factors and to simulate natural dynamics at canopy scale.

In this study we describe a novel automatic, low-cost system based on 12 open chambers able to measure instantaneous CO_2_ and H_2_O gas exchange and environmental conditions at canopy level. The system is flexible and allows to mimic different light conditions, either static or dynamic, allowing a good characterization of canopy photosynthesis comparable to field data. To our knowledge, few other growth chamber systems have this ability to mimic natural environmental conditions and have been described systematically including prices of the components, allowing a user-friendly reproduction of the system [34].

## A new low-cost and scalable whole plant gas exchange system

### Description of the system

The system (DYNAMISM, acronym for DYNAMIc photoSynthesis Measurements) we describe here is composed of twelve 0.54 m^3^ commercial growth chambers (60 × 60 × 150 cm; Secret Jardin, model Dark Dryer). The inlet ambient air is sucked into each chamber by a Blauberg inline mixed flow fan (diameter: 10 cm; flowrate: 102 m^3^ h^−1^) from a 4.5 m^3^ buffer chamber (150 × 150 × 200 cm; Secret Jardin, model Dark Street DS150). The buffer is needed to keep inlet CO_2_ and H_2_O concentrations as stable as possible during measurements and to control air temperature and humidity inside the growth chambers using an air conditioner. The inlet flow rate is measured at each chamber using a miniature air flow transmitter (E + E Elektronik, model EE671) placed before the inline fan and can be easily regulated by opening/closing the holes at the top and at the side of the chamber. The overpressure created inside each chamber by the flow fan avoids possible CO_2_ leakage or contamination during the measurements. Each air flow transmitter was calibrated against a reference mass flow meter before setting up the system (E + E Elektronik, model EE776; Additional file 1: Figure S1).

**Fig. 1:**
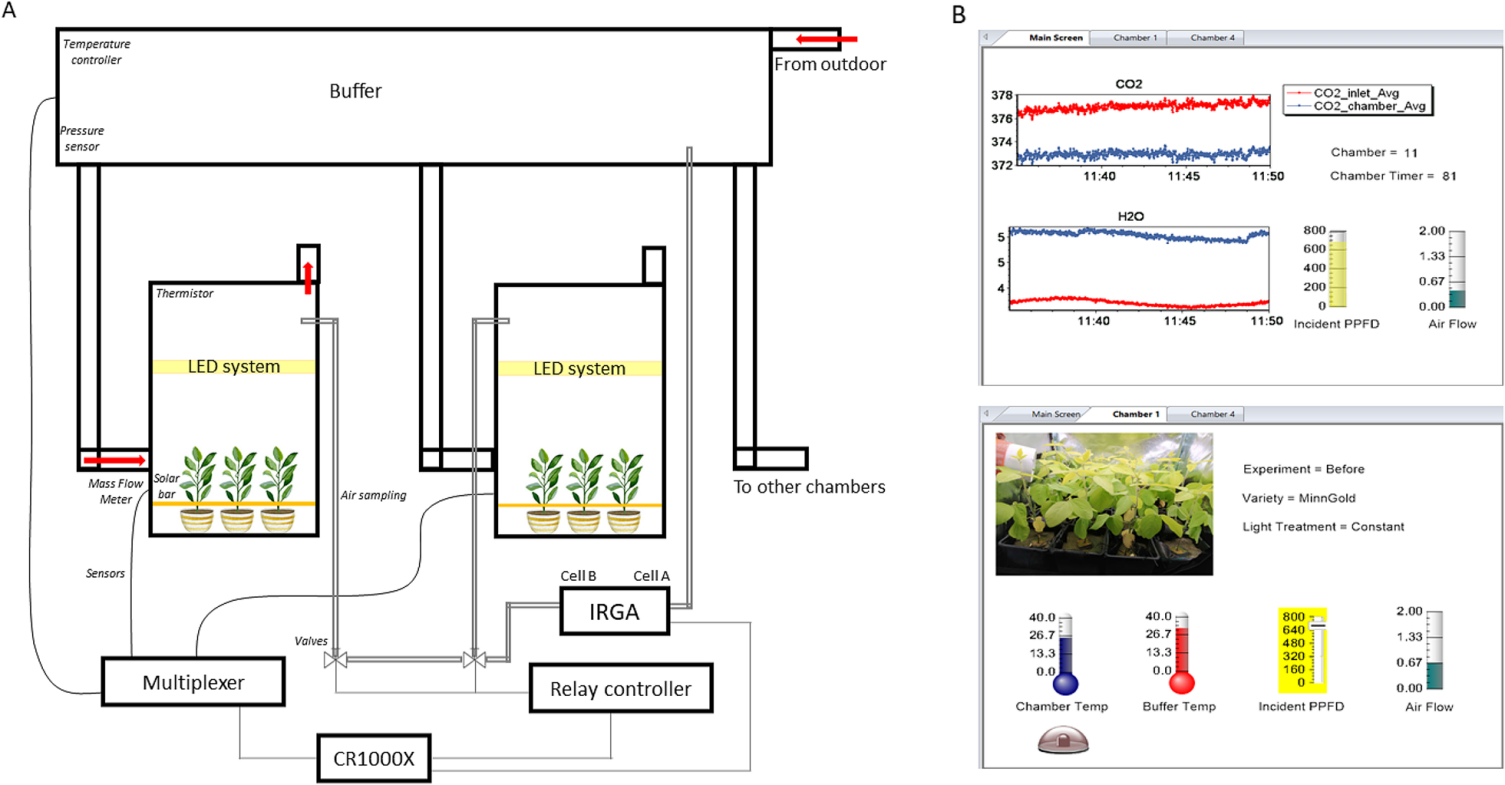
**A** Schematic representation of DYNAMISM. The 12 chambers (not all represented here) are connected to the bigger chamber that acts as a buffer. The buffer is itself connected to outdoor and has an air conditioning inside to keep the temperature and humidity more stable, and a pressure sensor. The air flows from the buffer to the chambers. Air is sampled within each chamber and analysed by the Licor-7000 (IRGA). Each chamber is equipped with a LED system, a mass flow meter to measure the inlet flowrate, a solar bar, a thermistor and an aquarium pump placed at the top chamber. Chamber sampling and data acquisition is made through a CR1000X datalogger which itself controls a multiplexer and a relay controller (SDM CD16-AC). **B** Example of the control of the CR1000X output variables through the RTMC software. In this case, in the main screen are shown the CO_2_ and H_2_O changes in real-time in the sampled chamber, as well as other environmental parameters. Then in each chamber the desired parameters can be monitored, here we have set an alarm for chamber temperatures higher than 40° and a slider input to change incident PPFD

Air temperature inside each chamber is measured using a thermistor (Measurement Specialties, Inc., model 10K3A1 Series 1) placed above the LEDs, while inlet air pressure is measured at the inlet of the main pipeline using an integrated pressure sensor (Freescale Semiconductor, Inc., model MPX4115A). A schematic representation of the system is reported in Fig. 1: the main pipeline starting from the buffer is made up of pipes with a diameter of 20 cm; chamber connecting pipes are 10 cm in diameter; the pipes connecting the buffer to outside the lab (outdoor) are 30 cm in diameter.

Instantaneous net canopy CO_2_ flux (A; µmol CO_2_ m^−2^ s^−1^) and instantaneous evapotranspiration (E; mol H_2_O m^−2^ s^−1^) are measured as differences in CO_2_ (μmol CO_2_ mol^−1^) and H_2_O (mmol H_2_O mol^−1^), respectively, in the air stream flowing through each chamber using a LI-7000 gas analyzer (Licor, USA) in differential mode. Inlet CO_2_ and H_2_O concentrations (i.e. concentration inside the buffer) are measured by pumping the air through a LI-840 gas analyzer and then to LI-7000 Cell A (reference). The outlet CO_2_ and H_2_O concentrations (i.e. concentration at the top of each chamber) are measured by pumping the air to the LI-7000 Cell B (sample) using an aquarium pump placed inside each chamber (Hailea, model ACO9602; flow rate: 7.2 l min^−1^). Reference and sample CO_2_ and H_2_O concentrations, air temperature and air pressure are recorded by a datalogger (CR1000X, Campbell Scientific, USA) by parsing the digital output of the LI-7000.

The sequential sampling of air inside the chambers is electronically controlled by the CR1000X through a 16 channel AC/DC controller (SDM CD16-AC, Campbell Scientific, USA), which stimulates each of the twelve 24 V solenoid valves connected to the aquarium pumps placed inside each chamber. Sampling frequency among the chambers, as well as sampling duration for each chamber, can be set by the user. A thirteen valve was connected to the main inlet within the buffer chamber allowing a periodic matching between Cell A and Cell B of the LI-7000. Such a matching is recommended in order to compensate for any differences in the two optical paths besides concentration differences. Outlet CO_2_ and H_2_O concentrations are thus corrected in post-processing and fluxes recomputed.

**Fig. 2:**
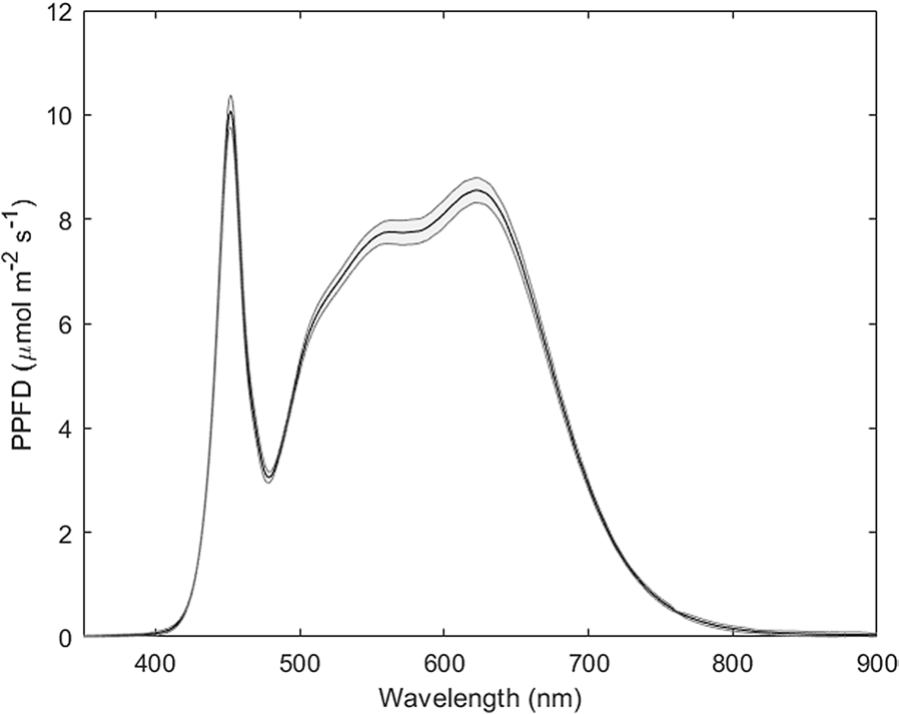
Light spectrum of the LED panels measured with FLoX at 10 cm distance (constant PPFD at 1876 ± 30 μmol m^−2^ s^−1^). The solid line is the mean, the grey shadow represents mean ± standard deviation (n = 6)

Each chamber is equipped with a 60 × 60 cm light system made up of 17 separate LED strips (Samsung SMD5630 “H-POWER", 185 W, 140 LED m^−1^, CRI90, Natural White, 4000 K). The light spectrum of the LEDs was measured using a fluorescence box (FloX, JB Hyperspectral Devices, Germany), and it well simulates the solar spectrum between 400 and 700 nm (Fig. 2). LEDs can be moved up and down inside the chambers depending on canopy height, and light intensity within each chamber is independently controlled by the CR1000X through a Modbus to voltage output converter (4E + Embedded Solutions, model DAT3028). The dimmer regulates the voltage signal (0–10 V) which determines the photosynthetic photon flux density (maximum PPFD = 1876 μmol m^−2^ s^−1^ at 10 cm distance when the number of LED strips per chamber is maximized).

The CR1000X can simulate daily solar radiation profile after the user sets the latitude and the longitude by computing solar elevation angle and knowing maximum PPFD or can simulate a fixed daily profile after the user chooses a fixed day of the year. Moreover, the user can simulate periodic light fluctuations around the hourly value by deciding the fluctuating range and the fluctuation period.

Transmitted radiation is measured using solar bars placed horizontally at the bottom of the canopy. Each bar is made of eight photodiodes in parallel (model S1087-01, Hamamatsu Photonics, Japan) with a 100 Ω resistance and was calibrated against a reference quantum sensor (Li-190R, Licor, USA) before setting up the system.

Finally, in Table 1 we report the list of all the major parts of the system, their technical specification and prices. The overall system cost is 5,000 euro (only 417 euro per chamber), without considering the reference sensors for calibrations, and the analyzers (LI-840 and LI-7000). One of the strengths of DYNAMISM is that any number of chambers is possible in the multiplexer mode, thus allowing to have a high number of replicates with a limited cost; nevertheless, if only a multiplexer is used, it will go in a repeated cycle.

**Table 1:** Description and technical specifications of all the system’s components

Sensor	Model and Manufacturer	Technical Specification	Prices	Sources
Growth chamber	Secret jardin—Dark street	60 × 60x150 cm (12 small chambers) 150 × 150x200 (buffer chamber)	84.4 €*12	https://www.idroponica.it/growbox-c-22/secret-jardin-s-311/dark-street-ds-secret-jardin-36855.html
LED	Samsung LED strip 5630 “H-POWER"	185 W; 140 LED/m SMD5630; Natural white: 4000 K 5-m length	106.4 € *12	https://store.ledpro.it/prodotti-led/strisce-led/strisce-led-linea-elite/striscia-led-5630-h-power-5-metri-185w-140-led-m-smd5630-samsung-bianco-naturale-4000k.html
Flowmeter for air flux	EE671—Miniature Air Flow transmitter—E + E electronica	Measuring range: 0–5 m/s; 0–10 m/s; 0–20 m/s Response time: 4 s	177.1 € *12	https://eu-shop.epluse.com/collections/air-velocity/products/355065
Flowmeter for scrubbing	SFM4100—Sensirion	Digital gas flow meter for gases	187 €	https://www.sensirion.com/en/flow-sensors/mass-flow-meters-for-high-precise-measurement-of-gases/mass-flow-meter-for-medical-gas-measurements/
Ventilator	Tube In-line fans—Blauberg ventilatoren	Diameter: 10 cm Energy Supply: 220 V AC Maximum air flow:102 m^3^/h	17.1 € *12	https://www.idroponica.it/cavo-alimentazione-200cm-con-spina-schuko~1146.html
Air pump	Hailea ACO9602	Pump speed: 7.2L/min	14.7 € *12	https://www.amazon.it/Pompa-dAria-Regolabile-Hailea-ACO9602/dp/B01GO80XE4 (not available in idroponica at the moment)
Pressure sensor	MPX4115—freescale semiconductor	Integrated Silicon Pressure Sensor	20 €	https://www.nxp.com/docs/en/data-sheet/MPX4115.pdf

Prices and companies (webpages) are also listed. All prices are indicated excluding VAT

### Gas exchange calculations

E (in mol H_2_O m^−2^ s^−1^) and A (in µmol CO_2_ m^−2^ s^−1^) are computed according to the following equations:

1 $$E=ai{r}_{flow}\times \frac{{H}_{2}{O}_{chamber}-{H}_{2}{O}_{in}}{S\times (1000-{H}_{2}{O}_{chamber})}$$

2 $$A=ai{r}_{flow}\times \frac{C{{O}_{2}}_{chamber}-C{{O}_{2}}_{in}}{S}-C{{O}_{2}}_{chamber}\times E$$

where ${H}_{2}{O}_{in}$ and ${{CO}_{2}}_{in}$ are the H_2_O (in mmol H_2_O mol^−1^) and CO_2_ (in μmol CO_2_ mol^−1^) concentrations within the buffer chamber (inlet) and ${H}_{2}{O}_{chamber}$ and ${{CO}_{2}}_{chamber}$ are the concentrations in each chamber; ${air}_{flow}$ is the air flux entering the chamber (mol s^−1^) and S is the chamber area (0.36 m^2^). We adopted the micro-meteorological convention to indicate CO_2_ uptake (net photosynthesis, negative value) and release (respiration, positive value). Air flow from the miniature air flow transmitter is converted from m s^−1^ (flow) to mol s^−1^ (air_flow_) according to the equation:

3 $$ai{r}_{flow}=\frac{flow\times {S}_{tube}\times P}{R\times ({T}_{chamber}+273.15)}$$

where S_tube_ is the tube area (0.10 × 0.10 m^2^), P is the inlet air pressure (Pa) and T_chamber_ is the air temperature inside the chamber (°C) and R is the universal constant of gases (8.3144598 m^3^ Pa K^−1^ mol^−1^).

### Performance and accuracy of DYNAMISM

Before testing the system with a real plant canopy, we simulated six different photosynthesis levels (A_sim_; μmol CO_2_ m^−2^ s^−1^) at five different air flux velocities (from 0.73 to 2.73 m s^−1^) in order to assess its performance and accuracy. We did this by using a tube filled with soda lime connected to a pump (Hailea ACO9602) and placed inside one of the chambers. By doing so we were directly scrubbing the air (i.e. removing CO_2_) within the chamber and we were able to calculate the exact flux of simulated photosynthesis according to the equation:

4 $${A}_{sim}=1000\times \frac{scru{b}_{flux}\times P}{R\times ({T}_{chamber}+273.15)}$$

where scrub_flux_ is the scrub’s pump speed (l s^−1^) measured using a flowmeter (Sensirion SFM4100), P is the air pressure (constant at 101,300 Pa), T is air temperature (°C) and R is the universal constant of gases (8.3144598 m^3^ Pa K^−1^ mol^−1^). The pump was turned on for 10 min at a first level of scrub’s pump speed (0.02 l s^−1^), then the scrub’s pump speed was increased at the second target velocity for another 10 min, and so on for all the six levels of simulated photosynthesis. When the maximum level of scrub flux (pump speed = 0.2 l s^−1^) was reached, the same procedure was applied from the highest value to the lowest. Final measured net CO_2_ fluxes were calculated from the system’s acquired data for the last 60 s of each step according to Eq. 2. When comparing all the five pump flux velocities, we expect that the steady state is reached faster at higher fluxes without affecting the steady state itself. In order to compare measured values at different speed of the scrub pump, we normalized the data by multiplying the ΔCO_2_ values for $Sx/\left({S}_{0}-Sx\right)$ where $Sx$ is the CO_2_ scrubbed flux and S_0_ is the CO_2_ flux at time 0; then we further rescaled the data through a min–max normalization. As expected, the results of these tests clearly show that higher the air flux, faster the steady state is reached (Additional file 1: Figure S2).

We also simulated five respiration levels (R_sim_) by injecting pure CO_2_ inside the chambers at two different air flux velocities (0.71 and 1.71 m s^−1^), following the same procedure (steps) described above for photosynthesis, using a gas mass flow controller for low flow rates (Bronkhorst, model F-201CV-100_RAD-00-Z). R_sim_ was computed according to the following equation:

5 $${R}_{sim}=\frac{C{{O}_{2}}_{injected}\times P}{S\times ({T}_{chamber}+273.15)\times R}$$

where CO_2injected_ is the CO_2_ injected flux (ml CO_2_ s^−1^), P is air pressure (101,300 Pa), S is chamber area (0.36 m^2^), T_chamber_ is chamber temperature (°C) and R is the universal constant of gases (8.3144598 m^3^ Pa K^−1^ mol^−1^).

**Fig. 3:**
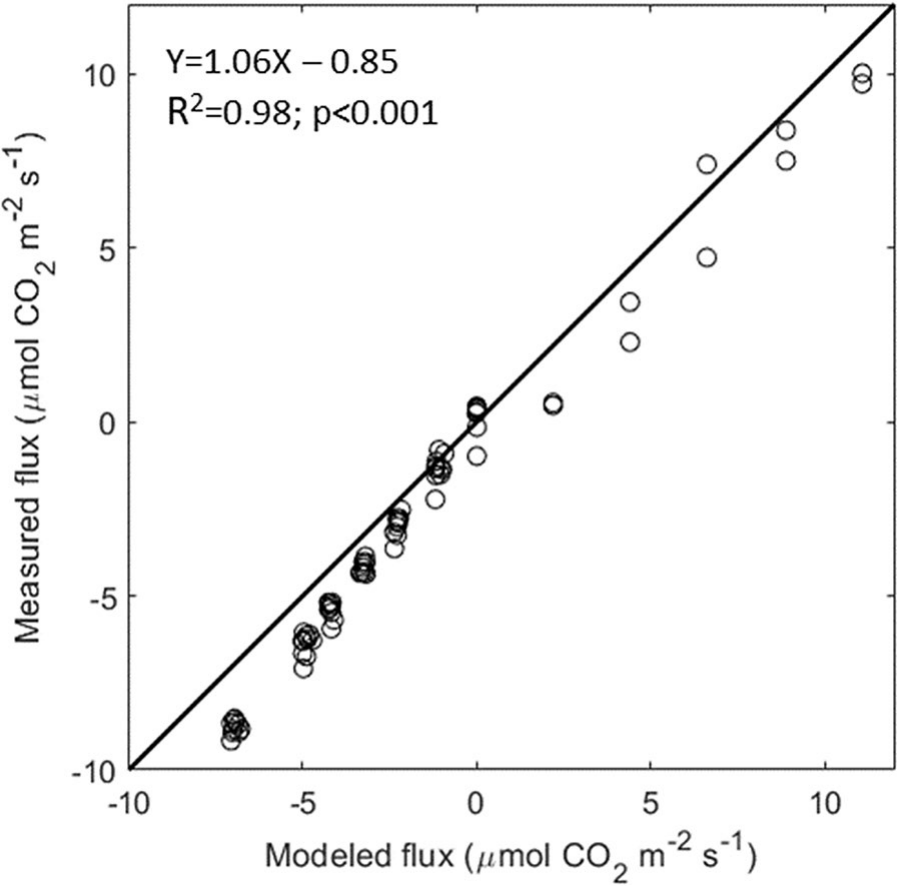
Preliminary test results: measured CO_2_ fluxes after scrubbing inlet CO_2_ (negative values) or after injecting pure CO_2_ (positive values) versus modeled fluxes calculated using Eqs. 4 and 5 for photosynthesis and respiration, respectively

The accuracy of DYNAMISM was finally assessed using a simple linear regression relating the measured values of CO_2_ after scrubbing the air or injecting pure CO_2_ with the values simulated with Eqs. 4 and 5. The system slightly overestimated CO_2_ fluxes (+ 7%, not significant) over the range from − 10 to 10 μmol CO_2_ m^−2^ s^−1^ (slope: 1.06; intercept: -0.85; R^2^ = 0.98; p < 0.001; Fig. 3).

### Performance evaluation through a mass balance model

To further assess DYNAMISM accuracy, we performed a comparison among the measured ΔCO_2_ values (${{CO}_{2}}_{in}$ – ${{CO}_{2}}_{chamber}$), obtained after scrubbing the air or injecting pure CO_2_, with a physical model based on a mass balance approach: the change in CO_2_ concentration with time inside the chamber depends on the CO_2_ entering the chamber from the buffer (${{CO}_{2}}_{in}$ in ppm) at a certain flux (F in mol s^−1^) minus the CO_2_ consumed by photosynthesis or released by respiration ($Sx$ in µmol CO_2_ s^−1^) and the internal concentration within the chamber (${{CO}_{2}}_{chamber}$ in ppm). Therefore, the physical model can be described by the following differential equation:

6 $$\frac{{dCO_{{2_{{chamber}} }} }}{{dt}} = \frac{{F \cdot CO_{{2_{{in}} }} - Sx}}{V} - \frac{{F \cdot CO_{{2_{{chamber}} }} }}{V}$$

By integrating this differential equation and by assuming perfect mixing within the chamber, the following equation is obtained:

7 $$\Delta CO_{2} = ~\frac{1}{F} \cdot \left( {Sx + \left( {S_{0} - Sx} \right) \cdot e^{{ - \frac{F}{V}\left( {t - t_{0} } \right)}} } \right)$$

where S_0_ is the CO_2_ flux at time 0, V is the chamber volume (19.3 mol) and ${t}_{0}$ is the delay due to chamber dimension (in seconds).

Solving Eq. 7 for $Sx$ results in:

8 $$Sx = \frac{{\Delta CO_{2} \cdot F}}{{1 - e^{{ - \frac{F}{V}\left( {t - t_{0} } \right)}} }} - \frac{{S_{0} \cdot e^{{ - \frac{F}{V}\left( {t - t_{0} } \right)}} }}{{1 - e^{{ - \frac{F}{V}\left( {t - t_{0} } \right)}} }}$$

In order to understand the error associated to $Sx$ measurements due to errors in the measured variables ($F$,${S}_{0}$, ${t}_{0}$ and ${\Delta CO}_{2}$), we made a sensitivity analysis. The total error was then computed according to Jordan and Sewell [13] by considering the partial derivatives of $Sx$ per each measured variable:

9 $$T = \sqrt {\left( {\frac{{\partial Sx}}{{\partial F}}\bar{F}} \right)^{2} + ~\left( {\frac{{\partial Sx}}{{\partial S_{0} }}\overline{{S_{0} }} } \right)^{2} + \left( {\frac{{\partial Sx}}{{\partial \Delta CO_{2} }}\overline{{\Delta CO_{2} }} } \right)^{2} + ~\left( {\frac{{\partial Sx}}{{\partial t_{0} }}\overline{{t_{0} }} } \right)^{2} }$$

where $\stackrel{-}{F}$, $\overline{{S_{0} }} ,~\overline{{\Delta CO_{2} }}$ and $\stackrel{-}{{t}_{0}}$ indicate the range in parameters for which the partial derivative is computed being $\stackrel{-}{F}=\left[0.2-0.6\right]$ mol s^−1^,$\stackrel{-}{{S}_{0}}=[0-5]$ µmol CO_2_ s^−1^, $\stackrel{-}{{t}_{0}}=\left[0-100\right]$ s $,~\overline{{\Delta CO_{2} }} = \left[ {0 - 10} \right]$ ppm.

**Fig. 4:**
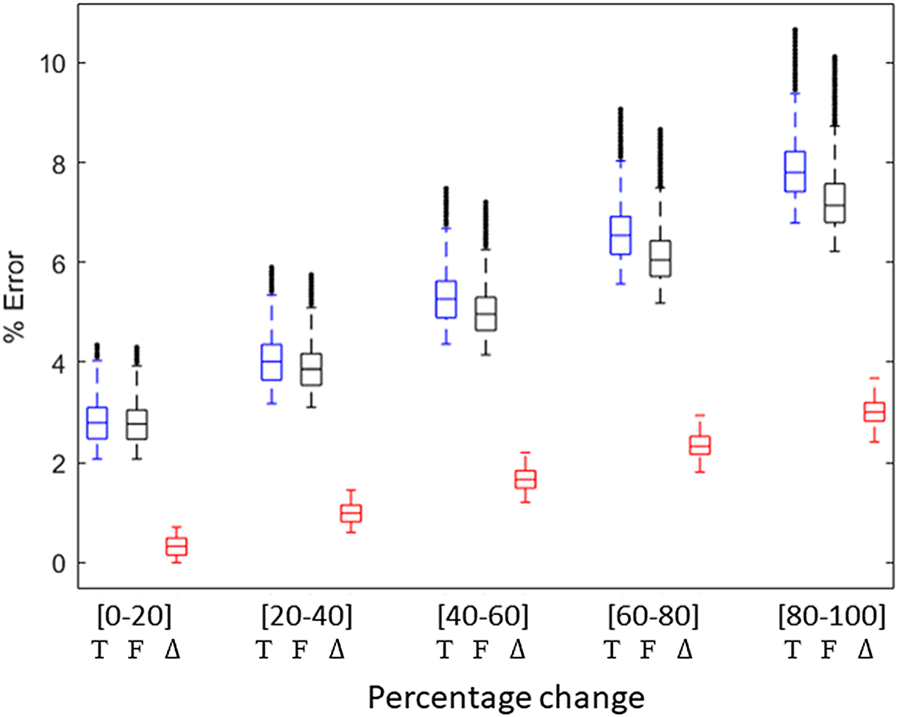
Total percentage error (T) and percentage errors due to changes in air flux velocity (F) and in $\Delta {\mathrm{C}\mathrm{O}}_{2}$ ($\Delta$) calculated from the partial derivation of Eq. 8. The parameter changes (x axis) are shown as normalized values (i.e. percentage change [0–100]) but the actual ranges of parameters are: F = [0.2: 0.6] mol s^−1^ and $\Delta {\mathrm{C}\mathrm{O}}_{2}$=[0: − 10] ppm. The boxplots show the aggregated values for all 12 chambers

In Fig. 4, we reported the total error (T) and the errors related to F and $\Delta {CO}_{2}$ only, as those due to ${S}_{0}$ and ${t}_{0}$ were smaller than 1% and thus negligible. According to our sensitivity analysis, the major source of error in the measurements of CO_2_ fluxes with DYNAMISM is related to F, especially at the highest air flux velocity (0.6 mol s^−1^), underlying the need to use an accurate flowmeter to assess it.

### Correct for delays

**Fig. 5:**
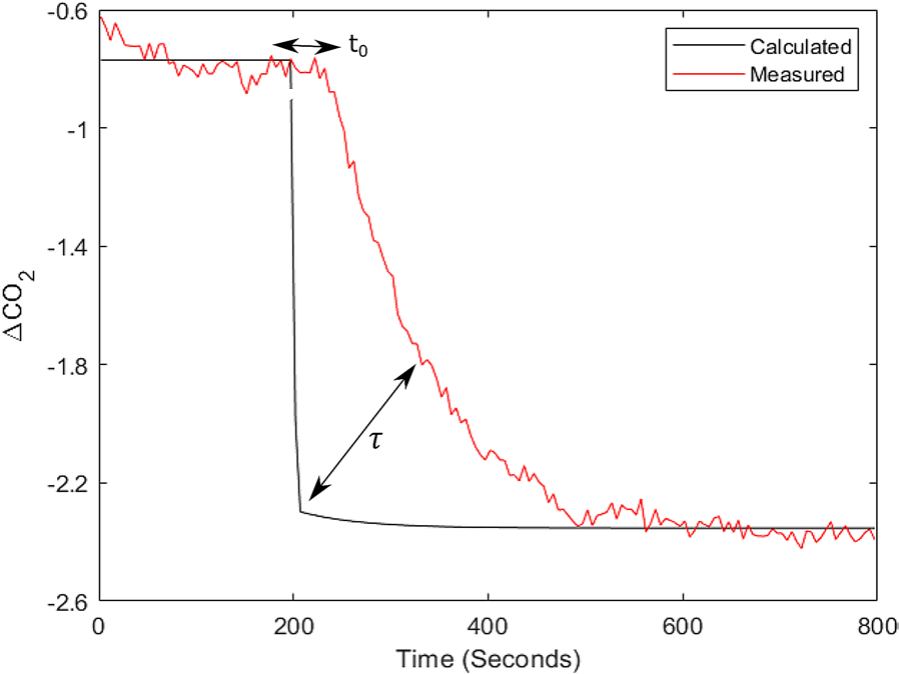
Example of the scrubbing of CO_2_ with an air flux velocity (F) of 0.34 l s^−1^ (red line, measured data). The black line indicates the $\Delta \mathrm{C}{\mathrm{O}}_{2}$ corrected for the delay and residence time (τ and t_0_, respectively). The lines represent 5 s averaged values

The model described in Eqs. 7 and 8 allows to mathematically compute the delay of the measuring system (${t}_{0}$) due to the lengths of the tubes and the volume of the chamber, and the residence time (τ, unitless), which applies in case of no perfect mixing. In fact, in such last case, Eqs. 7 and 8 need to be changed by adding τ to the exponent value, which reads as $\tau (t-{t}_{0})$. By first fitting the $\Delta {CO}_{2}$ calculated according to Eq. 7 with the τ correction to the measured data and then using the fitted parameters values to compute $Sx$ based on Eq. 8 (i.e. perfect mixing, no τ correction), it is possible to estimate τ and t_0_. If this procedure is repeated at least once per day in a chamber by scrubbing/injecting CO_2_, it is possible to have an estimate of both $\tau$ and ${t}_{0}$ and correct the measured data for the delays (Fig. 5). In fact, as the structure of the canopy itself changes over time affecting the mixing within the chamber, this procedure allows having a daily correction of the data taking into account the delays.

### Estimating steady state $\Delta \boldsymbol{C}{\boldsymbol{O}}_{2}$

**Fig. 6:**
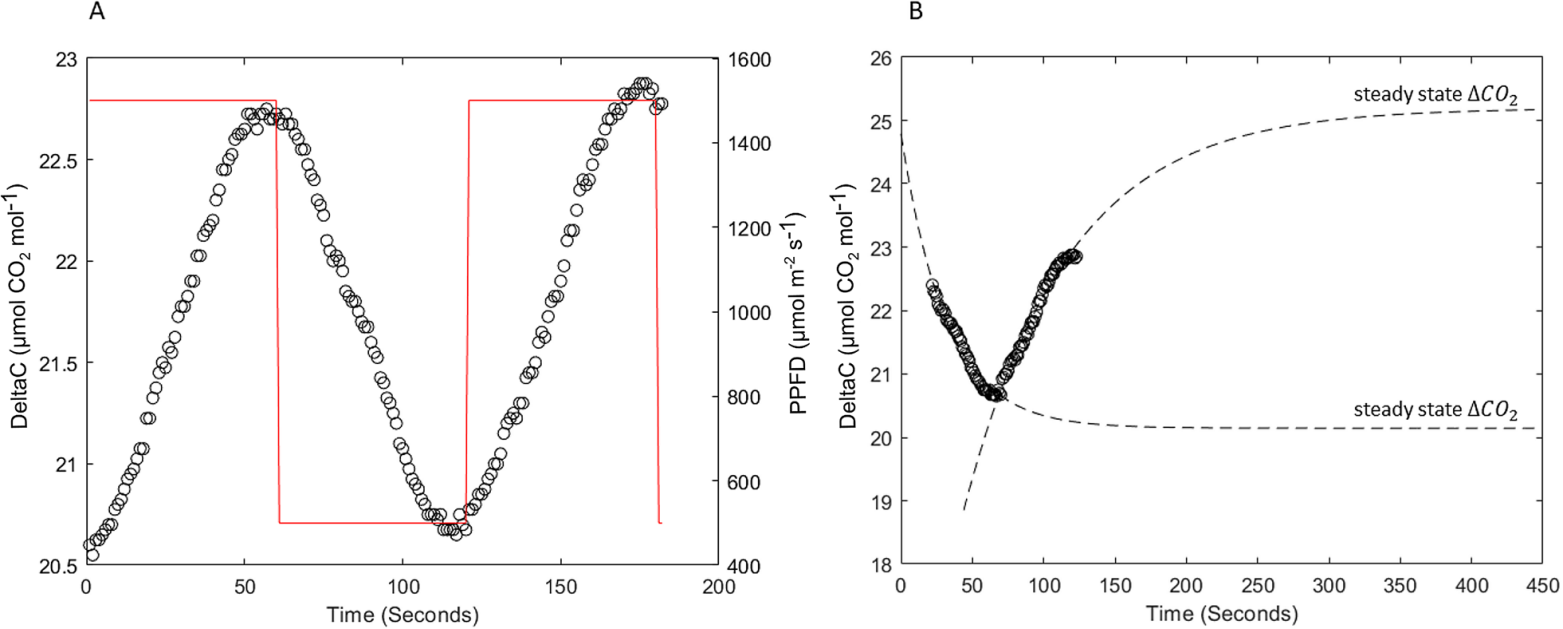
**A** $\Delta \mathrm{C}{\mathrm{O}}_{2}$ changes due to fluctuations in light intensity. **B** Fitting of the data in A (in the range 60 to 180 s) through Eq. 7 and estimation of steady state $\Delta \mathrm{C}{\mathrm{O}}_{2}$

The described modelling framework also allows to determine steady state fluxes from unsteady state data by fitting Eq. 7 with the τ correction to $\Delta {CO}_{2}$ measured values. To test this, we grew a soybean variety inside the chambers with fluctuating light conditions and measured the changing canopy photosynthetic rates. As the light was fluctuating (with a period of 2 min) it determined a continuous change in the $\Delta {CO}_{2}$ due to canopy carbon assimilation (i.e. photosynthesis). Since fluctuations were very frequent, the measured values never reached steady state (Fig. 6A). The fitting procedure though allowed to have an estimate of the steady state values by fitting unsteady state $\Delta {CO}_{2}$ values (Fig. 6B), showing that steady state is reached only after about 300 s (as also evident from Fig. 5).

### Quantifying whole plant gas-exchange under fluctuating conditions

To test the accuracy of DYNAMISM in real conditions, we used a commercial soybean variety (Eiko, Asgrow, USA). Plants were sown in 96 pots (13 × 13 × 18 cm) with siliceous sand in order to have an inert substrate and to zeroing heterotrophic respiration (Rh). We used six chambers for the experiment, and we placed 16 pots chamber^−1^.

In three chambers, the LED system was set to simulate a fixed daily profile (June 21^st^) in Udine, Italy (latitude: 46.07 N; longitude: 13.23 E) with a maximum PPFD of 1000 µmol m^−2^ s^−1^ at noon (non-fluctuating light treatment, NF). In the other three chambers (fluctuating light treatment, F), light was fluctuated ± 50% with a period of 120 s around the hourly value measured in NF. By doing this, plants grown either in fluctuating or non-fluctuating light received the same total light intensity throughout the day. According to the light curve reported by Sakowska et al. [40], these fluctuations at midday (500–1500 µmol m^−2^ s^−1^) fall within the saturated range of the curve, therefore the highest values of light are saturating. It is than predictable that the cumulative average value (1000 µmol m^−2^ s^−1^) would entail a higher C assimilation than the cumulative fluctuating values. This though is not the case when the oscillations of light fall into the linear range of the light curve, as in the first (and last) hours of the day. In this case, we expect the average value of light to be translated into a similar accumulation of CO_2_.

LEDs were manually moved up inside the chambers as canopy grew thus to be at a constant distance of 13 cm above the plants throughout the experiment.

Each chamber was sampled for 290 s and A was calculated as average between 110 and 290 s thus to not consider the tube’s purging after chamber switch (t_0_ = 110 s). The matching procedure with the thirteenth valve was done every hour in order to compute the difference in CO_2_ and H_2_O concentration among the cell A and B of the LI-7000, thus correcting the data based on this value. Measurements were run for four weeks during which plants were regularly watered with the addition of a Hoagland solution twice per week (Table 2).

**Table 2:** Nutrients (mL) for a 100% Hoagland solution

Components	Stock (g/L)	mL stock/30L
Macro-nutrients		
1 M KNO_3_	101	150
1 M Ca(NO_3_)_2_ 4H_2_O	236	150
Fe-EDTA	15	30
2 M MgSO_4_ 7H_2_O	123	120
1 M KH_2_PO_4_	136	30
Micro-nutrients		
H_3_BO_3_	2.86	30
MnCl_2_ 4H_2_O	1.81	
ZnSO_4_ 7H_2_O	0.22	
CuSO_4_ 5H_2_O	0.08	
H_2_MoO_4_ H_2_O	0.09	

For Soybean we used a half strength solution: nutrients for 30L diluted in 60L of distilled water per week. pH of 6.47

At the end of the experiment, we harvested four plants per chamber. Leaf area was measured using a LI-3000 (Licor, USA), stem and leaves were separated from roots and these lasts were gently washed to remove sand. Leaves, stems and roots were then dried at 70 °C for 48 h and then weighted. Because of the inert substrate used in the pots (no heterotrophic respiration, Rh), the measured CO_2_ flux corresponds to net primary production (NPP = A) instead of net ecosystem production (NEP = NPP – Rh), allowing a direct comparison between the cumulative A flux (gC m^−2^) at the end of the experiment and the total produced biomass (i.e. total dry weight; g m^−2^) by assuming a C content of 46.8% [40].

**Fig. 7:**
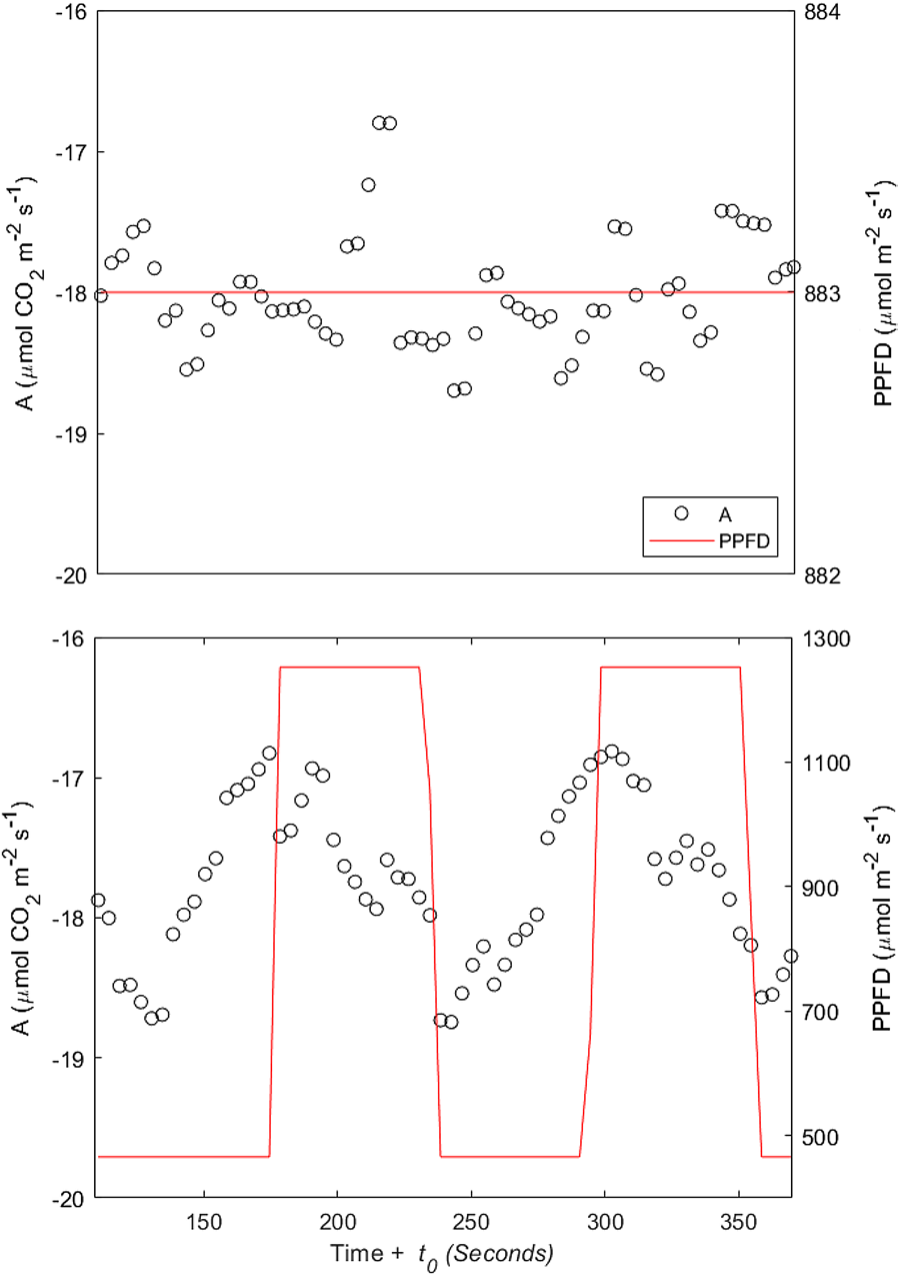
Soybean CO_2_ fluxes in non-fluctuating (above) and fluctuating light conditions (below). Data are instantaneous measurements during one session (25^th^ July at 10:00 am). Red lines represent photoflux density (PPFD), dots represent CO_2_ fluxes. CO_2_ fluxes data are corrected for the delayed response (t_0_ = 110 s). The lines represent 4 s averaged values. More negative values of A at higher PPFD values corresponds to higher photosynthesis (micro-meteorological convention)

Considering the response time due to the dimension of the chambers (t_0_ = 110 s) the system was clearly able to detect instantaneous changes in A related to light fluctuations, while it measured stable A in non-fluctuating light conditions (Fig. 7).

**Fig. 8:**
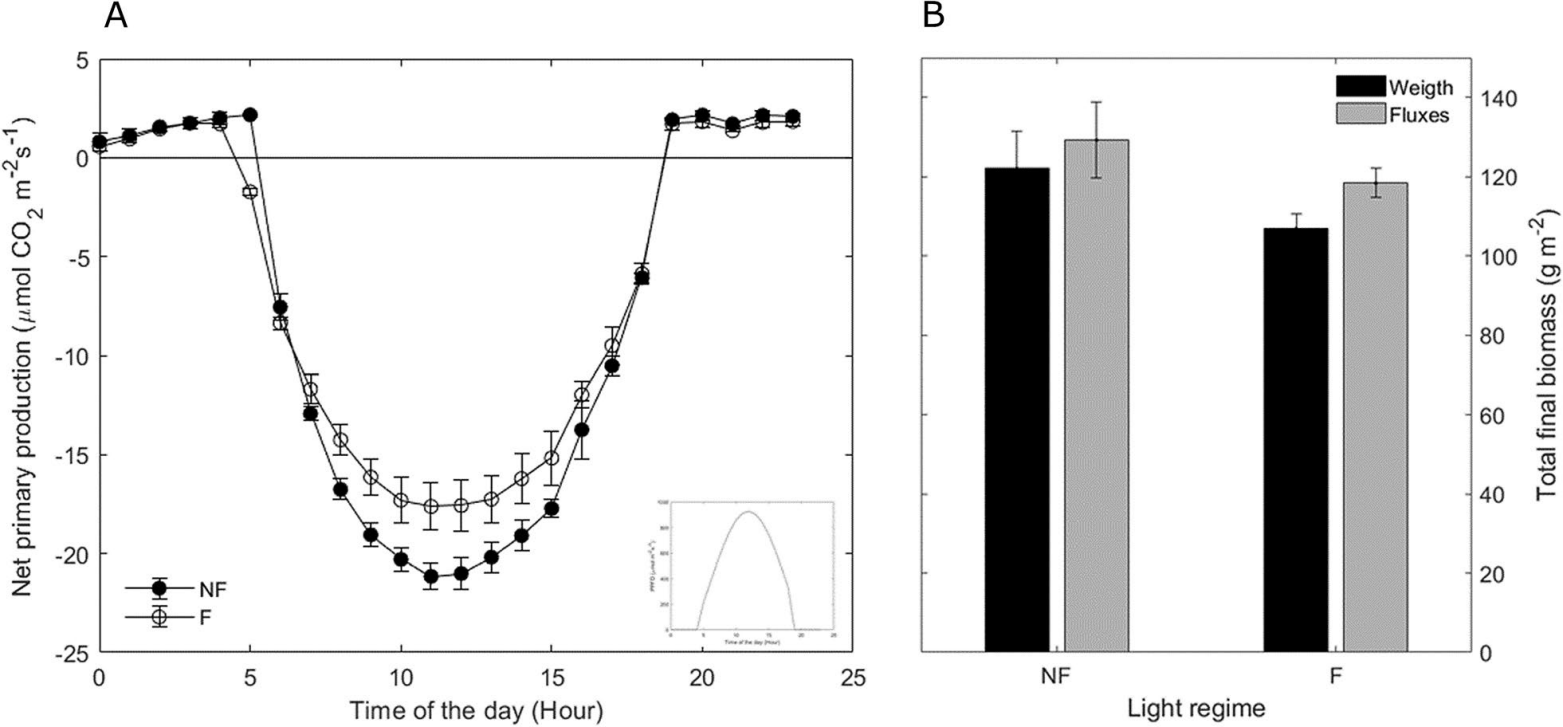
**A** Daily course of net primary production (NPP) measured five weeks after sowing. Closed and open symbols are fluctuating (F) and non-fluctuating (NF) light conditions, respectively. In the inner panel, the daily course of PPFD is reported. Negative NPP values denote C uptake following the micro-meteorological convention. **B** Total final biomass derived from fluxes and from plant dry weights at harvest for the two considered treatments. Any significant difference was found at harvest. Vertical bars are standard error (n = 3)

On an hourly basis, the system responded as expected: from a positive CO_2_ flux during night (respiration) to a maximum net uptake (negative flux) at midday with a small variability among chambers (Fig. 8A). At the end of the experiment, cumulative fluxes were not significantly different from the total plant dry biomass measured at harvest (Fig. 8B), confirming the applicability of DYNAMISM to measure canopy CO_2_ fluxes.

## Discussion and conclusions

Several approaches have been used in the literature to obtain reliable measurements of CO_2_ assimilation. Leaf level data are mainly reliable but the scaling to plant or canopy scale is rather difficult. On the other hand, canopy scale methods exist and can capture CO_2_ exchange dynamics at bigger scales but suffer from several weaknesses [1, 36]. Therefore, to overcome these issues, many growth chamber systems have been developed in the last decades, but most of them lack the ability to measure dynamic environmental conditions, such as those generally occurring in the field, and/or are extremely expensive. We demonstrated that the main strength of DYNAMISM relies on its accuracy and stability (Figs. 3, 4 and 5), on the possibility to accurately estimate the response time and to correct for the intrinsic delays of the system (Fig. 6) and to determine steady state fluxes from unsteady state measured values (Fig. 7). Thus, it is able to efficiently capture the effect of fast fluctuating light on instantaneous CO_2_ gas exchanges (Fig. 8).

Finally, DYNAMISM can be used for several applications: different plant canopies can be monitored thanks to the flexibility in the software and to the dimension of the chambers, allowing to answer relevant biological questions.

Even though we focused our attention in this paper on light fluctuations, DYNAMISM could be used in the future also to simulate other dynamic environmental conditions such as temperature, humidity and CO_2_ concentration with some simple upgrades and at a limited cost. Thus, it can be potentially used to induce abiotic stresses, by simulating, for example, drought conditions, high light conditions (inducing photo-inhibition) and high environmental CO_2_ levels. Moreover, as a future development, we think to couple DYNAMISM with real-time fluorescence measurements to investigate photochemistry and the primary reactions of photosynthesis in dynamic environments as well as to use it for photosynthesis phenotyping [18].

## Supplementary Information

**Additional file 1: Figure S1.** Calibration curve of the miniature air flow transmitters. The straight line represents the overall regression (all sensors). **Figure S2**. Changes in ΔCO_2_ at different air flux levels when scrubbing the air with soda lime. The data show all ramps (i.e. different pump speeds) normalized from 0 to 1 and averaged every 20 seconds.

## Data Availability

The datasets used and/or analysed during the current study are available from the corresponding author on reasonable request.
