# Supplementary material for: A low-cost automated growth chamber system for continuous measurements of gas exchange at canopy scale in dynamic conditions
Source: Plant Methods. 2021 Jun 30;17:69. doi: 10.1186/s13007-021-00772-z (PMC8243713; doi:10.1186/s13007-021-00772-z)

**Additional File 1**

***Figure S1.*** *Calibration curve of the miniature air flow transmitters. The straight line represents the overall regression (all sensors).*


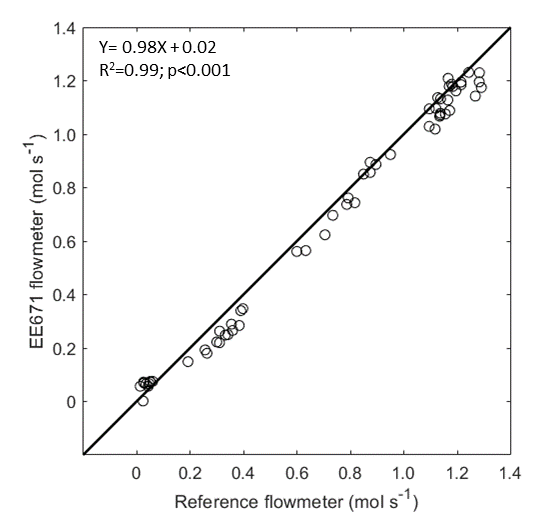


***Figure S2****. Changes in ΔCO_2_ at different air flux levels when scrubbing the air with soda lime. The data show all ramps (i.e. different pump speeds) normalized from 0 to 1 and averaged every 20 seconds.*


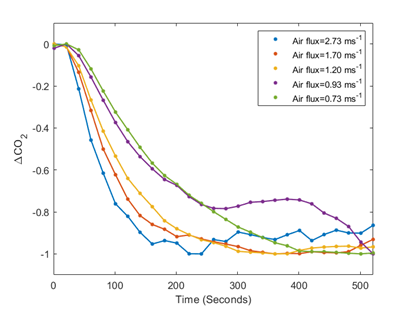

Supplement: Supplementary file 1 — Additional file 1: Figure S1. Calibration curve of the miniature air flow transmitters. The straight line represents the overall regression (all sensors). Figure S2. Changes in ΔCO2 at different air flux levels when scrubbing the air with soda lime. The data show all ramps (i.e. different pump speeds) normalized from 0 to 1 and averaged every 20 seconds. [file 13007_2021_772_MOESM1_ESM.docx]
